# Intracellular shuttling of a Drosophila APC tumour suppressor homolog

**DOI:** 10.1186/1471-2121-5-37

**Published:** 2004-09-30

**Authors:** Adam Cliffe, Julius Mieszczanek, Mariann Bienz

**Affiliations:** 1 MRC Laboratory of Molecular Biology, Hills Road, Cambridge, CB2 2QH, UK; 2 EMBL, Meyerhofstr. 1, D-69117 Heidelberg, Germany

## Abstract

**Background:**

The Adenomatous polyposis coli (APC) tumour suppressor is found in multiple discrete subcellular locations, which may reflect sites of distinct functions. In Drosophila epithelial cells, the predominant APC relative (E-APC) is concentrated at the apicolateral adherens junctions. Genetic analysis indicates that this junctional association is critical for the function of E-APC in Wnt signalling and in cellular adhesion. Here, we ask whether the junctional association of E-APC is stable, or whether E-APC shuttles between the plasma membrane and the cytoplasm.

**Results:**

We generated a Drosophila strain that expresses E-APC (dAPC2) tagged with green fluorescent protein (GFP-E-APC) and we analysed its junctional association with fluorescence recovery after photobleaching (FRAP) experiments in live embryos. This revealed that the junctional association of GFP-E-APC in epithelial cells is highly dynamic, and is far less stable than that of the structural components of the adherens junctions, E-cadherin, α-catenin and Armadillo. The shuttling of GFP-E-APC to and from the plasma membrane is unaltered in mutants of Drosophila glycogen synthase kinase 3 (GSK3), which mimic constitutive Wingless signalling. However, the stability of E-APC is greatly reduced in these mutants, explaining their apparent delocalisation from the plasma membrane as previously observed. Finally, we show that GFP-E-APC forms dynamic patches at the apical plasma membrane of late embryonic epidermal cells that form denticles, and that it shuttles up and down the axons of the optic lobe.

**Conclusions:**

We conclude that E-APC is a highly mobile protein that shuttles constitutively between distinct subcellular locations.

## Background

The Adenomatous polyposis coli (APC) protein is an important tumour suppressor in the colonic epithelium [1]. A key function of this highly conserved protein is to antagonize Wnt signalling, by constitutively downregulating the transcriptional activity of β-catenin/Armadillo, a key effector of the Wnt signalling pathway [2]. Loss of this function is thought to be critical in the initiation of colorectal tumorigenesis as it causes a transcriptional switch in the intestinal epithelium towards actively dividing crypt progenitor cells [3-5]. APC proteins are highly conserved among vertebrates and flies, and flies encode two APC proteins with overlapping roles in Wnt signalling during development [6,7].

However, APC proteins have additional functions in connection with the actin and microtubule cytoskeletons that appear to be separate from their function in controlling Wnt signalling [8,9]. One of these functions is a role of APC in facilitating cellular adhesion, as indicated by studies in Drosophila tissues [10] and in mammalian colorectal cancer cells [11]. This function in cellular adhesion is likely to be conferred by the subcellular pool of APC protein that is associated with adherens junctions (AJs) in Drosophila [12,13] and in polarised mammalian cells [14]. The mechanism by which APC facilitates cellular adhesion is unknown.

In order to explore this mechanism, we asked whether Drosophila E-APC (also called dAPC2) might have a structural role at AJs. If so, E-APC would be expected to be stably associated with AJs, similarly to the structural components of the adhesive complex. As in mammalian epithelia [15,16], the main functional components of this complex in Drosophila are the transmembrane protein E-cadherin, a calcium-dependent trans-membrane adhesion molecule, and the catenins (Armadillo and α-catenin) that link E-cadherin to the actin cytoskeleton at the cytoplasmic side [17-22]. We thus conducted photobleaching experiments on live embryos expressing E-APC or structural AJ components tagged with green fluorescent protein (GFP) [23-25], to compare their relative mobility. These experiments revealed that GFP-E-APC is less stably associated with AJs than their structural components. We also found that GFP-E-APC is remarkably mobile in neurons.

## Results and discussion

**Figure 1:**
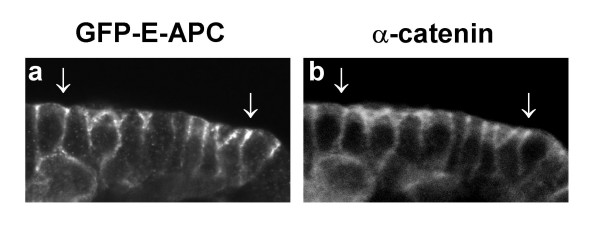
Association of GFP-E-APC with AJs of embryonic epithelial cells. Side view of epidermis of a ~6 hour old embryo expression ubiquitous GFP-E-APC, stained with antibody against (**a**) GFP and (**b**) α-catenin to mark the apicolateral AJs; note the co-incidence of GFP-E-APC and α-catenin staining (arrows).

**Figure 2:**
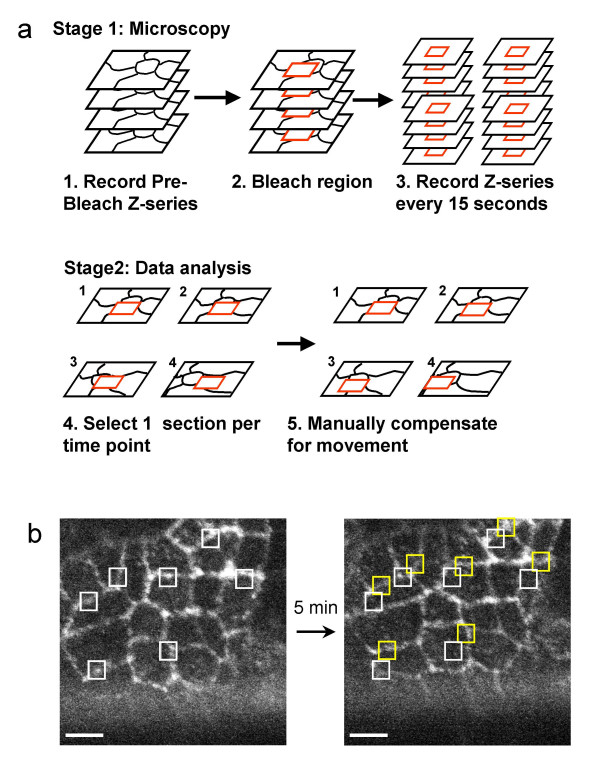
FRAP protocol for the analysis of live Drosophila embryos expressing GFP-E-APC. (**a**) Sketch of the microscopy and data analysis used to determine the mobility of GFP-E-APC in epithelial cells of live embryos. (**b**) Consecutive face-on views of a live ~6 hours old embryo (stage 11) expressing GFP-E-APC, with squares marking specific sections of the AJs at cell interfaces; the set of yellow squares in the right-hand image illustrate the cell shape changes that took place during the 5 minutes between the two optical sections shown. White bar in this and subsequent figures, 5 μm.

We used the GAL4 system to express GFP-E-APC throughout the embryo, and found that its subcellular distribution is very similar to that of endogenous E-APC in fixed embryos. In particular, GFP-E-APC is concentrated underneath the plasma membrane in apicolateral regions of embryonic epithelial cells (Fig. 1a; Fig. 2b). These regions form the zonula adherens (ZA) which contains the AJs [26]; they can also be visualised with antibody staining against α-catenin [27] (Fig. 1b), and we observe a remarkably close coincidence of GFP-E-APC and α-catenin. Similar results were obtained by Akong at el. who examined the same GFP-E-APC transgene in the embryo [7], and who also showed that GFP-E-APC is distributed similarly as endogenous E-APC in larval neuroblasts [28].

**Figure 3:**
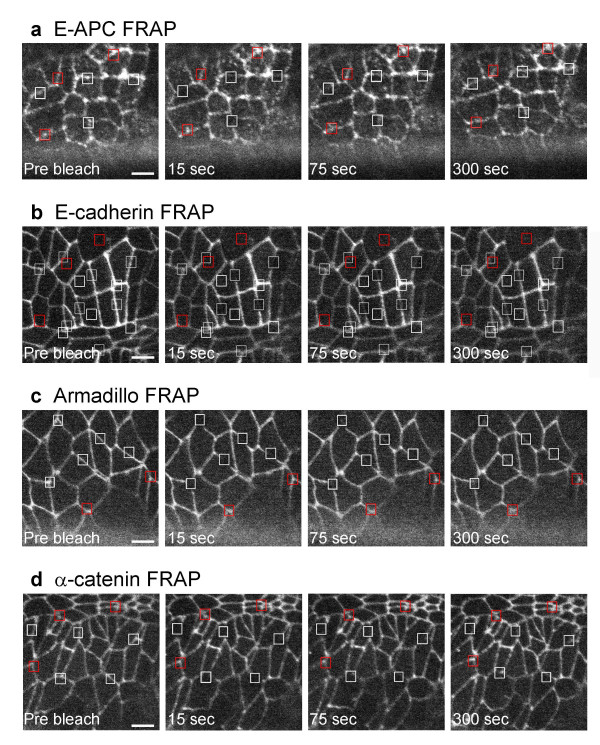
FRAP of GFP-E-APC and GFP-tagged AJ proteins in early embryonic epithelial cells. Face-on views of live ~6 hours old embryos (stage 11) expressing (**a**) GFP-E-APC, (**b**) E-cadherin-GFP, (**c**) Armadillo-GFP, (**d**) α-catenin-GFP, with white squares marking sections of cell interfaces that were bleached, and red squares marking unbleached control sections. Pre-bleaching images are shown on the left; subsequent images on the right show recovery of fluorescence within white squares 15, 75 and 300 seconds after bleaching [see additional file 1].

**Figure 4:**
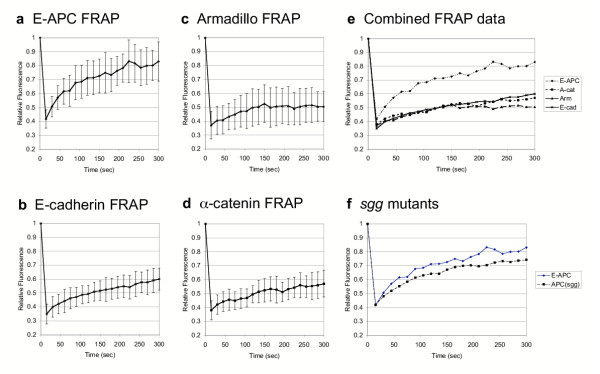
Quantitative evaluations of FRAP experiments. Plots of the relative fluorescence in white squares compared to grey squares in Fig. 3 as a function of time; error bars indicate the range of values from >12 different experiments. (**a-d**) FRAP of individual GFP-tagged proteins in wild-type embryos, as indicated; (**e**) combined data of (**a-d**); (**f**) Comparison of FRAP of GFP-E-APC in wild-type and *sgg *mutant embryos (note that we were unable to distinguish between null mutant and paternally rescued embryos; however, the rescue activity of the paternal allele is minimal as both types of embryos are highly abnormal).

Next, we conducted fluorescence recovery after photobleaching (FRAP) experiments in live embryos, to examine how stably GFP-E-APC is associated with adherens junctions. We bleached the fluorescence in a defined square centred over the junctional region of an epithelial cell with a short laser pulse, and examined the recovery of the fluorescence within this square over time (Fig. 2a). This revealed a relatively fast rate of recovery of most of the fluorescence within a few minutes (Fig. 3a) [see additional file 1]. Quantitative analysis shows that nearly 80% of the initial fluorescence is recovered within ~220 seconds (with a half-time value of ~60 seconds) (Fig. 4a,4e). This indicates that the association of E-APC with the ZA is dynamic rather than stable. The fluorescence recovery observed in these FRAP experiments could be due to movement of E-APC between the cytoplasm and the plasma membrane, but also to sideways movement along the plasma membrane (see also below).

We also conducted FRAP experiments with structural AJ components, namely E-cadherin-GFP, Armadillo-GFP and α-catenin-GFP. In these cases, we can only recover a small fraction of the initial fluorescence within the time frame of the experiment (Fig. 4a,4b,4c,4d,4e; note that these experiments cannot be extended beyond ~6 minutes, due to the extensive cell shape changes during this developmental stage). Furthermore, the rate of recovery is slower than that observed with GFP-E-APC, with estimated half-times of >3 minutes (α-catenin-GFP and of E-cadherin-GFP; Fig. 4b,4d,4e). This also appears to be true for Armadillo-GFP (Fig. 4c,4e), though we cannot estimate its half-time of recovery with confidence, given that its fluorescence levels are considerably lower than that of the other GFP-tagged protein examined in this study.

We conclude that E-APC is significantly more mobile than the structural AJ components. This suggests that E-APC shuttles either within the cortex, along the zonula adherens, or that it shuttles from the cytoplasm to the plasma membrane (as previously proposed; [29]). Interestingly, the observed rates of recovery of GFP-E-APC were much slower than the estimated rate of free diffusion (e.g. [30]; the rate of recovery of GFP alone was <10 seconds, i.e. too fast to be measured by our experimental setup). This suggests that the movements of GFP-E-APC are primarily determined by the kinetics of its binding to ligands. One of these could be Axin which associates with E-APC in Drosophila cells to from large dot-like structures [31]. Similarly, Axin associates with APC in mammalian cells to form large molecular weight protein complexes [32]. Our observations argue against a structural role of E-APC in cellular adhesion. However, they are consistent with a catalytic role of E-APC in facilitating cellular adhesion, for example by maintaining the junctional pool of Armadillo [10,29,33]. In support of this, recent evidence suggests that there is rapid exchange of β-catenin within the junctional cadherin complex, and that APC is required for this process [34].

**Figure 5:**
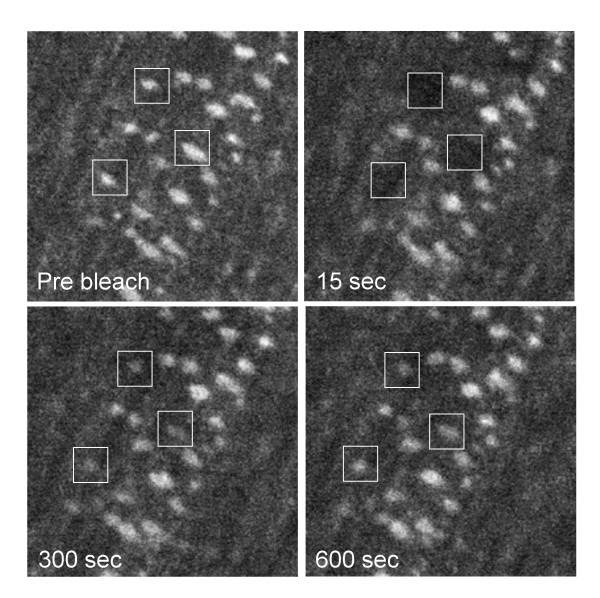
FRAP of GFP-E-APC patches in late embryonic epithelial cells. Face-on views of ~17 hours old embryo (stage 17) expressing GFP-E-APC, showing patches of GFP-E-APC at the apical plasma membrane of epidermal cells forming denticles (pre-bleach and subsequent images labelled as in Fig. 3). Note the fast recovery of the fluorescence in these patches after photobleaching.

In late embryonic stages, GFP-E-APC forms striking patches underneath the apical plasma membrane of epidermal cells that are in the process of forming denticle extrusions (Fig. 5). These striking 'pre-denticle' patches are also seen in embryos stained with antibody against E-APC, and overlap with actin patches [25]. They may thus represent an actin-dependent association of E-APC as that seen in the cortex of earlier epithelial cells and at the ring canals between nurse cells within the egg chambers [25,33]. FRAP experiments revealed that the presence of GFP-E-APC in these pre-denticle patches is also dynamic, with an estimated half-time of fluorescence recovery of 200–300 seconds (Fig. 5). Again, E-APC is therefore unlikely to have a structural role in these patches.

It has been reported that E-APC and Armadillo are required for anchoring mitotic spindles in the cortex of dividing blastoderm cells in the early Drosophila embryo [25]. We cannot measure the kinetics of GFP-E-APC association with the cortex in these early embryonic cells, because of insufficient expression levels at this stage. However, assuming that these kinetics do not change radically during embryonic development, our observations from the later embryos (Fig. 3,4,5) suggest that E-APC has a catalytic role in capturing microtubules in the cellular cortex, rather than providing a structural tether [25].

**Figure 6:**
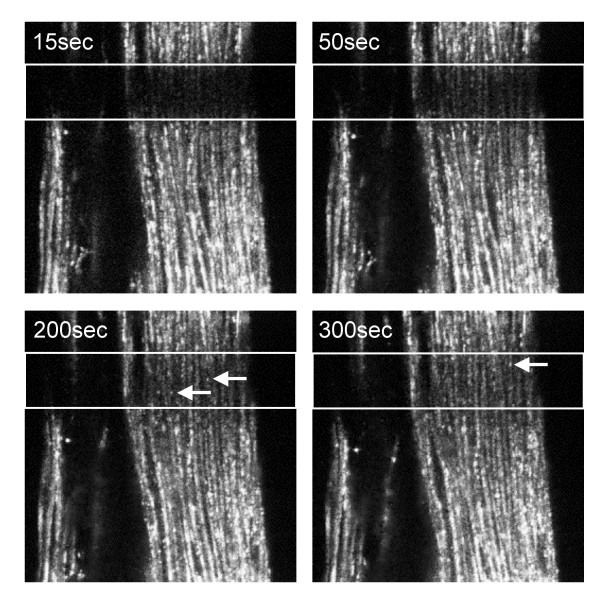
FRAP of GFP-E-APC in the larval optical stalk. Optical sections through the optical stalk of a third instar larva, before and after photobleaching, showing fluorescent puncta in individual axons, and the reappareance of these puncta (arrows) from both sides of the bleached areas within minutes [see additional file 2]. Width of bleached strip, 6 μm.

We also expressed GFP-E-APC in eye imaginal discs, to examine its subcellular distribution within a larval epithelial sheet. We thus noticed striking puncta of green fluorescence within the axons of the optic stalk that connects these discs to the larval brain (Fig. 6). These puncta resemble the E-APC/Axin-GFP dots that we observe in embryonic cells [31] and in these axons (not shown), and also the E-APC/Armadillo dots that Peifer and colleagues observed in early embryos [25]. They may thus represent the Axin destruction complex [31]. We performed FRAP experiments, bleaching a 6 μm wide strip perpendicularly across the axons and monitoring the recovery of the fluorescence into the bleached section. This revealed that the GFP-E-APC puncta are remarkably dynamic: they re-appear within the bleached area within a minute, with an estimated half-time of ~100 seconds (Fig. 6) [see additional file 2]. Many of these puncta seem to re-appear from other focal planes, so we cannot be absolutely certain that they represent movement of existing puncta. However, some of the puncta re-appearing in the bleached area can clearly be traced as moving puncta within the same focal plane (e.g. see isolated axon, left-hand side of [additional file 2]). The movement of these GFP-E-APC puncta may be due to tracking (e.g. along microtubules), although we cannot see uni-directionality of movement (i.e. the movement appears to be up and down the axons). The movement we observe in these axons is somewhat reminiscent of that observed with a GFP-tagged truncation of Xenopus APC that misses its C-terminus (thus resembling the overall structure of E-APC): this truncation, despite lacking the putative microtubule-interacting domain within its C-terminus, forms large puncta that can track along microtubules in Xenopus tissue culture cells [35].

**Figure 7:**
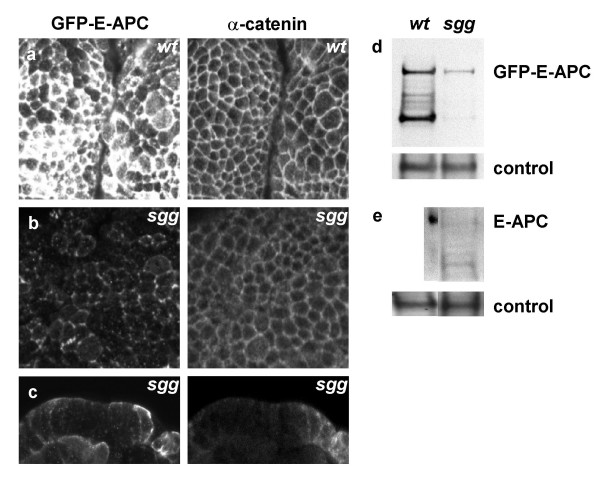
Destabilisation of E-APC in *sgg *mutant embryos. (**a, b**) Face-on and (**c**) side views of ~14 hours old embryos (stage 16), fixed and co-stained with antibodies against GFP and α-catenin as indicated, revealing junctional association of GFP-E-APC in (**a**) wild-type and (**b, c**) *sgg *mutant embryos (similar in *sgg *null and paternally rescued embryos, see also Fig. 4f). (**d, e**) Western blots of hand-picked 10–16 hours old wild-type and *sgg *mutant embryos (~100 embryos per lane), probed with antibodies against (**d**) GFP or (**e**) E-APC, and α-tubulin as internal controls. Note that the levels of GFP-E-APC and of endogenous E-APC are much reduced in *sgg *compared to wild-type embryos (*sgg *mutants represent a 1:1 mixture of *sgg *null and paternally rescued embryos). The lower bands in upper panels (**d, e**) correspond to breakdown products of GFP-E-APC and E-APC, respectively; their occurrence varies somewhat between preparations.

GSK3 is inhibited during Wnt signalling [2], and GSK3 mutants in Drosophila (*shaggy*/*zeste white3*, or *sgg*, mutants) therefore mimic constitutive and sustained Wingless signalling [36]. The normal level of Wingless signalling in the embryonic epidermis does not appear to change the subcellular distribution of bulk E-APC protein [12], although it does cause a re-location of Axin-GFP/E-APC complexes to the plasma membrane [31]. However, a reduction of cortical E-APC has been observed in early *sgg *mutant embryos [12,25]. Likewise, in older *sgg *mutant embryos, the levels of membrane-associated GFP-E-APC are also noticeably reduced (Fig. 7a,7b,7c). However, this does not appear to be due to a change in mobility of GFP-E-APC since the kinetics of fluorescence recovery between wild-type and *sgg *null mutant embryos were comparable (Fig. 4f). Instead, it is due to a reduction of the overall E-APC protein levels in these mutants: Western blot analysis of 2–16 hour old embryos revealed that the total levels of GFP-E-APC protein were much lower in *sgg *mutant embryos compared to the wild type (Fig. 7d). The same is true for endogenous E-APC whose levels are also substantially reduced in *sgg *mutants (Fig. 7e). This indicates that *sgg *is required for the stability of E-APC protein, and it suggests that sustained Wingless signalling may destabilise E-APC. Similarly, phosphorylation by GSK3 is required for the stability of mammalian Axin, a functional binding partner of APC [37], and the levels of Drosophila Axin in embryos are also reduced after prolonged Wingless signalling [38]. Destabilisation of the main components of the Axin complex (Axin and APC) during Wnt signalling may be a positive feedback mechanism resulting in the amplification of the signalling level.

The subcellular distribution of E-APC and its accumulation at the adherens junctions is unchanged in other mutants of the Wingless signalling pathway (including *wg*, *axin*, *dsh *and signalling-defective *arm *mutants; [31]; F. Hamada, X. Yu and M. B., unpublished observations). We thus did not expect any of these mutants to affect the shuttling behaviour of GFP-E-APC to and from the plasma membrane. In support of this, preliminary FRAP experiments indicated that the kinetics of fluorescence recovery are unaffected in *dsh *null mutant embryos (not shown). Taken together with our results from the *sgg *mutants, this suggests that the kinetic association of GFP-E-APC with the plasma membrane is unaffected by Wingless signalling.

## Conclusion

Our FRAP experiments provided evidence that E-APC is a cytoplasmic shuttling protein whose association with the adherens junctions is highly dynamic. The speed of its shuttling to and from the plasma membrane appears to be constitutive and does not require GSK3 activity. The dynamic association of E-APC with the plasma membrane is consistent with a catalytic role of E-APC, and argues against a structural or tethering role in the cell cortex.

## Methods

### Fly strains

Fly lines transformed with UAS.GFP-E-APC (full length E-APC tagged with GFP at its N-terminal end, inserted into pUAST [39]) were generated by R. Rosin-Arbesfeld (see also [7,28]). The GAL4 driver lines arm.GAL4 and GMR.GAL4 (FlyBase) were used to express GFP-E-APC throughout the embryonic epidermis [31] and in the larval eye disc, respectively. All fly strains were cultured at 25°C.

*zw3*^*M11-1 *^and *dsh*^*v26 *^mutant embryos lacking maternal and zygotic gene function were generated as described [40]. We did not detect any differences in the subcellular localisation of GFP-E-APC or α-catenin between zygotic null and paternally rescued *sgg *mutants (identified with an RFP-marked X chromosome [41]). For Western blot analysis, 10–16 hours old wild-type and *sgg *mutant embryos were hand-picked (from timed egg collections) under the dissecting microscope, and separated into GFP-positive and GFP-negative embryos; unfertilised embryos were discarded.

### Analysis of fixed embryos and Western blots

Antibody staining of fixed embryos and analysis by confocal microscopy were described previously [12]. The following primary and secondary antibodies were used: rabbit anti-E-APC [12], rabbit anti-GFP [14], rat anti-α-catenin [42]; goat anti-rat IgG Alexa Fluor 568, goat anti-rabbit IgG Alexa Fluor 488 (Molecular Probes).

The following primary and secondary antibodies were used for Western blotting: rabbit anti-E-APC [12]; mouse monoclonal anti-GFP IgG2a (Santa Cruz Biotechnology); mouse anti-α-tubulin (clone B-5-1-2, Sigma), as internal control; goat anti-mouse and anti-rabbit HRP IgG (Santa Cruz Biotechnology). The enhanced chemiluminescence (ECL) Western blotting system (Amersham) was used for detection [43].

### Live imaging of embryos

For live imaging, embryos were dechorionated in 50% bleach for 1–2 minutes and washed. Embryos were transferred to a moistened black filter (Schleicher and Schüll). Embryos were adhered to coverslips with heptane glue, made by mixing heptane and clear sellotape (Sellotape Ltd). Embryos were mounted in Voltalef oil (10S). For short term imaging (<30 minutes), embryos were mounted on a glass slide with small coverslips as supports. For longer term imaging, e.g. for bleaching of pre-denticle patches, embryos were mounted in oil and placed on Bio-foil gas permeable membrane (Sartorius Ltd) mounted on a perspex frame [44].

### Photobleaching of live embryos

FRAP experiments were performed using a Bio-Rad Radiance confocal microscope with a 40× NA 1.3 objective lense. Imaging was performed with a 488 nm argon laser at 5% laser power and the following confocal settings: iris at 4 mm, 50% gain, zoom 10, scan speed 500 lps, box size 512 × 512 pixels. These conditions were found to give minimal photobleaching over the observed time.

For each FRAP experiment, a pre-bleach image was recorded by selecting a focal plane and taking a Z-series, consisting of 3 0.5 μm steps either side of the desired focal plane (from -1.5 μm to +1.5 μm). The LaserSharp software was used to define several regions of interest (ROIs) for bleaching. A maximum of one bleach ROI was placed in any cell and several cells were always left unbleached. Typically, 3–5 ROIs were bleached in one field of view on one embryo. These regions were bleached at 100% laser power (scanning at 500 lps). 10 bleach scans were found to produce the best results for all constructs. After bleaching, a Z-series was recorded every 15 seconds for 5 minutes. At the time of these experiments, the LaserSharp software did not contain a function for performing this type of 4D bleaching experiment. This problem was overcome by manually switching between imaging and bleaching settings and manually saving pre bleach images and starting the time course. As a result of this, there was usually a 30–60 second delay between the pre-bleach image and the post-bleach images.

### Data analysis

Data sets were analysed with the Bio-Rad LaserPix software. For each time point, the total pixel intensity distribution was compared to the pre-bleach image to select the corresponding region. The two images were then compared by eye to confirm that they did correspond to the same focal plane. The coordinates for the bleach ROIs were used to accurately locate the bleach spots on the pre bleach image, and the mean fluorescence intensity for each ROI was calculated. Several equivalent sized ROIs were also placed on unbleached cells to measure any change in fluorescence due to photobleaching or movement.

To track movement of the cells, an acetate sheet was placed over the computer monitor and each ROI was marked on it as well as the shapes of the cells surrounding it. By aligning the sheet with the appropriate cell shapes, the ROI could be appropriately positioned for each time point. This process was used to position each ROI on the appropriate image for each time point.

Once all ROIs had been placed on the image, the mean fluorescence intensities were calculated for each ROI, and their positions were saved on a copy of the image (See Fig 2.1). Data was exported to Microsoft Excel for analysis. Relative fluorescence was calculated for each bleach area by dividing fluorescence at time (t) by pre-bleach fluorescence. The change in fluorescence was plotted on a graph with Excel. For each construct tested, the data from multiple bleach experiments from multiple embryos were averaged to give the approximate rate of recovery.

Data sets were discarded for any of the following reasons. First, if movement of the embryo in the Z axis took the sample outside the range of the Z-series in any time point. Second, if movement in the X/Y axis was sufficient to move significant numbers of the bleach boxes outside of the observed region. Third, if an ROI ever left the field of view, all data points for that ROI was discarded. Fourth, all data sets were discarded if the intensities of the control ROIs changed dramatically at any point in the experiment, or showed a large general increase or decrease.

Pre-denticle structures were bleached in a similar manner to junctional E-APC described above.

### Live imaging of the larval optic stalk

GFP-E-APC was expressed in eye imaginal discs by the GAL4 system, using the driver line GMR.GAL4 (described in FlyBase). Eye discs and brains were dissected from crawling third instar larvae in PBS. Eye discs were teased away from the brain and inverted to reveal the optic stalk. Whole disc/brains were mounted in a drop of PBS under a cover slip, supported by two smaller cover slips. Each disc was observed for no more than 30 minutes.

### Photobleaching of the larval optic stalk

FRAP experiments were performed using a Bio-Rad Radiance confocal microscope and Bio-Rad LaserSharp software, using the 100× NA 1.4 objective lens. A narrow strip was bleached across the whole field of view by adjusting the size of the scanning area. These experiments were performed before a FRAP program was available for LaserSharp so bleaching was performed manually, leading to somewhat variable intervals between each stage of the experiment. The region was bleached with the 488 nm line of an argon laser for approximately 20 scans. Time courses were recorded after each bleaching experiment for 5 minutes.

## Authors' contributions

A.C. developed and conducted most of the FRAP experiments; J.M. completed the analysis of GFP-E-APC in live and fixed *sgg *mutant embryos, including the Western blots; M.B. directed the study, helped with the microscopy and drafted the manuscript. All authors read and approved the final manuscript.

## Supplementary Material

Additional File 1 FRAP of GFP-E-APC in early embryonic epithelial cells. Example of a FRAP experiment of GFP-E-APC, as described in Figure 3. Click here for file

Additional File 2 FRAP of GFP-E-APC in the larval optical stalk. Example of a FRAP experiment of GFP-E-APC, as described in Figure 6. Click here for file
